# Antifungal resistance and stewardship: a call to action in Zambia

**DOI:** 10.11604/pamj.2023.45.152.41232

**Published:** 2023-08-08

**Authors:** Steward Mudenda, Billy Chabalenge, Maisa Kasanga, Webrod Mufwambi, Ruth Lindizyani Mfune, Victor Daka, Scott Kaba Matafwali

**Affiliations:** 1 Department of Pharmacy, School of Health Sciences, University of Zambia, Lusaka, Zambia,; 2 Department of Medicines Control, Zambia Medicines Regulatory Authority, Lusaka, Zambia,; 3 Zhengzhou University, College of Public Health, Zhengzhou, China,; 4 Department of Public Health, Michael Chilufya Sata School of Medicine, Copperbelt University, Ndola, Zambia,; 5 Clinical Research Department, Faculty of Infectious and Tropical Diseases, London School of Hygiene and Tropical Medicine, Keppel Street, London, United Kingdom

**Keywords:** Antimicrobial resistance, antifungal resistance, antifungal stewardship, antimicrobial stewardship, fungal infections, Zambia

## To the Editors of the Pan African Medical Journal

Fungi are known to cause superficial and invasive diseases, some of which are life-threatening [1]. While antifungal drugs have been instrumental in treating fungal infections caused by a variety of fungi [2], there is a rising concern with certain fungi species, notably *Aspergillus* species and *Candida* species which have evolved to resist the commonly used antifungals [3]. In recent years, there has also been a rise in non-albicans Candida species, such as *Candida glabrata, Candida krusei*, and *Candida tropicalis*, which have shown reduced susceptibility to commonly used antifungals [4]. Azole-resistant *Aspergillus fumigatus* has also been reported, which can lead to invasive infections with high mortality rates [5]. Antifungal Resistance (AFR) is a public health problem, that occurs when fungi develop resistance to antifungal drugs [3]. This phenomenon has been exacerbated by the often inappropriate use and misuse of antifungals in treating fungal infections [6]. Notably, these antifungals are prescribed empirically, and in most cases obtained without using prescriptions. Therefore, the inappropriate use of antifungal medications is a major contributor to the development of AFR [7]. This not only makes treatment more difficult but also increases the risk of morbidity and mortality [7]. In Zambia, much like antibiotics, antivirals, and antimalarials, most antifungal drugs are easily accessed without valid prescriptions. Additionally, these drugs are mostly prescribed empirically. We posit that due to a lack of data on AFR in Zambia, many patients, especially those with compromised immunity, have died from resistant infections. Our hypothesis is supported by studies globally that underscore the rising mortality due to fungal infections and AFR [3,5]. Addressing AFR necessitates a multifaceted strategy involving healthcare professionals and stakeholders across various sectors [8]. Antifungal stewardship (AFS) programs are paramount and promote the rational use of antifungals, and monitor their prescribing, dispensing and use [9]. The programs have demonstrated effectiveness in reducing inappropriate prescribing and irrational use of antifungal drugs, subsequently reducing patient mortality [10]. However, implementing AFS programs can be challenging due to limited resources and a lack of awareness of AFR among healthcare workers and the general public. To overcome these challenges, we need a coordinated effort from leaders, healthcare professionals, policymakers, patients, and other key stakeholders to promote AFS. The current Antimicrobial Stewardship (AMS) programs in Zambia focus on antibiotic resistance and stewardship, leaving AFR and AFS comparatively under-addressed. To this effect, the aims of the Zambia National Action Plan (NAP) on antimicrobial resistance (AMR) are also neglected. This oversight has the potential to cause an increase in morbidity and mortality due to fungal diseases and therefore necessitates urgent attention.

**Policy recommendations:** i) The Zambia National Public Health Institute (ZNPHI) should encourage all institutions and researchers involved in AMR research to extend their focus to AFR and AFS including adding it to the list of priority research areas by the National Health Research Authority (NHRA). Additionally, there is a need to support research and innovation by investing in antifungal drug development, and AFR patterns in different settings to guide treatment decisions. ii)The government through the Zambian Ministry of Health (MoH) should allocate funding to AFS that tackle AFR, especially since many opportunistic infections among immunocompromised patients are fungal in origin and may lead to patients succumbing to antifungal-resistance infections. iii) Healthcare authorities such as the ZNPHI and the Zambian MoH should formulate guidelines for antifungal prescribing and use. Such guidelines would aid healthcare workers to understand the prescribing patterns and use of antifungals. iv) National Antifungal Resistance Action Plan: Zambia should develop and implement a comprehensive National antifungal resistance action plan. This plan should be a collaborative effort involving key stakeholders from the healthcare, agricultural, and veterinary sectors employing a one health approach. It should include strategies for surveillance, infection prevention and control, AMS, and public awareness campaigns. v) Healthcare authorities such as the ZNPHI and the Zambian MoH should work towards establishing a national surveillance system for AFR and fungal infections. This system should track patterns of resistance, identify emerging hotspots, and facilitate the early detection of outbreaks. The data collected will be instrumental in guiding public health interventions. vi) Healthcare authorities such as the ZNPHI and the Zambian MoH should develop public health programs that can impart knowledge and awareness on AFR among the general public to combat the misuse and abuse of antifungal drugs.

## Conclusion

Despite Zambia's high burden of infectious diseases that compromise immunity and thus increase susceptibility to fungal infections, there is a lack of research on AFR and AFS. This glaring gap necessitates an urgent need to encourage research efforts focusing on antifungal use, prescribing patterns, dispensing, resistance patterns, and stewardship in the country. Comprehensive surveillance systems for monitoring AFR and fungal infections are essential to inform evidence-based policies and treatment guidelines.
